# Demonstration of microchimerism in pregnant sows and effects of congenital PRRSV infection

**DOI:** 10.1186/1297-9716-43-19

**Published:** 2012-03-16

**Authors:** Uladzimir U Karniychuk, Wander Van Breedam, Nadine Van Roy, Claire Rogel-Gaillard, Hans J Nauwynck

**Affiliations:** 1 Laboratory of Virology, Faculty of Veterinary Medicine, Ghent University, Ghent, Belgium; 2 Center for Medical Genetics, Ghent University Hospital, Ghent, Belgium; 3 INRA, Laboratory of Animal Genetics and Integrative Biology, Jouy-en-Josas, France

## Abstract

The presence of foreign cells within the tissue/circulation of an individual is described as microchimerism. The main purpose of the present investigation was to study if microchimerism occurs in healthy sows/fetuses and if porcine reproductive and respiratory syndrome virus (PRRSV) infection influences this phenomenon. Six dams were inoculated intranasally with PRRSV and three non-inoculated dams served as controls. Male DNA was detected in female fetal sera of all dams via PCR. Male DNA was also detected in the maternal circulation. Sex-typing FISH showed the presence of male cells in the female fetal organs and vice versa. PRRSV infection did not influence microchimerism, but might misuse maternal and sibling microchimeric cells to enter fetuses.

## Introduction, methods and results

The presence of small numbers of foreign cells within tissues or circulation of an individual is described as microchimerism [1]. Naturally acquired microchimerism refers to sibling (exchange of fetal cells in between siblings), maternal (presence of maternal cells within fetus) and fetal microchimerism (presence of fetal cells within mother) [2-16]. Fetal and maternal cell exchange is common in human and rodent pregnancy [1]. Both fetal and maternal microchimerism has been associated with different autoimmune disorders, pregnancy pathology and transplantation complications [1,17-19]. Otherwise, fetal cell differentiation in maternal tissues is presumably involved in tissue repair and protection against cancer [1]. Microchimerism may also play an important role in the physiology and pathology of pregnancy. However, at present no information is available on this phenomenon in swine.

Porcine reproductive and respiratory syndrome virus (PRRSV) is the cause of severe reproductive problems in sows [20]. The means by which PRRSV crosses the placental barrier remain unknown. Prior to fetal infection, PRRSV replicates in the endometrium and shows a very restricted tropism for Sn+/CD163+ macrophages [21,22]. Therefore, PRRSV might use susceptible cells as a vehicle to cross the uterine epithelium/trophoblast layers. Some pathological conditions during pregnancy, especially diseases that affect the placental environment, might influence fetal and maternal cell exchange. For instance, it has been observed that a mild inflammation caused by Pertussis toxin enhances cell migration through the murine placenta [23]. During replication in the endometrium, PRRSV causes apoptosis of infected and surrounding cells [22] and changes the expression of cellular receptors (increased Sn expression and change in the MHC class I and II expression) (not published results). Potentially, PRRSV-mediated changes in endometrial macrophages and/or in uterine epithelial/trophoblast layers may trigger events which influence the cell transmigration process. If a physiological cell exchange between mother and fetuses occurs in swine, it is interesting to examine if PRRSV infection influences it. By facilitating transplacental cell migration, PRRSV infection may favor cell-mediated PRRSV transfer from mother to fetuses. In contrast, PRRSV-mediated changes in maternal tissues may also inhibit transmigration processes, resulting in the protection of fetuses. Therefore, the main purpose of the present investigation was to study if microchimerism occurs in healthy sows/fetuses and if PRRSV infection influences this phenomenon.

Nine dams from a PRRSV-free herd were kept in isolation rooms. The experimental design is summarized in Table 1. Six dams were inoculated intranasally with 10^5 ^TCID_50 _type 1 PRRSV 07 V063 (GenBank No: GU737264) at 70 (dam I_70_) or 90 (I_90_-1, I_90_-2, I_90_-3, I_90_-4, I_90_-5) days of gestation in 4 mL of phosphate buffered saline (PBS) (2 mL in each nostril). Three non-inoculated dams were included in the study (Table 1). Blood was collected from all dams before and after PRRSV inoculation. The control animals were euthanized at 100 days of gestation. PRRSV-inoculated dams were euthanized at 10 days post-inoculation (I_70_, I_90_-1, I_90_-2, I_90_-3) or at 20 days post-inoculation (I_90_-4, I_90_-5) and uteri were removed. The uterine wall adjacent to every fetus was incised and fetal blood was collected from the umbilical cords. Blood was collected with individual disposable syringes and gloves were changed prior to every sampling. The gender of individual fetuses was recorded and lungs and liver of each fetus were collected and snap frozen. To confirm PRRSV infection, virus isolation and titration from maternal and fetal sera and PRRSV-specific immunofluorescence (IF) staining on frozen tissue sections were performed [22,24].

**Table 1:** Experimental design.

Dam	Parity	**PRRSV inoculation at... gd**^**†**^	Euthanized at... gd	Number of fetuses		
				
				Total	Female	Male
I_70_	4	70	80	11	7	4
I_90_-1	5	90	100	14	9	5
I_90_-2	1	90	100	14	5	9
I_90_-3	1	90	100	17	9	8
I_90_-4	1	90	110	12*	8	2
I_90_-5	1	90	110	16*	9	6
Con-1	1	-	100	13	7	6
Con-2	10	-	100	16	11	5
Con-3	6	-	100	15	7	8

^†^gd: gestation day; -: not inoculated. *Due to mummification, the gender determination in two and one fetus from dams I_90_-4 and I_90_-5, respectively, was not possible.

DNA was isolated from sera (200 μL) of fetuses and dams using a QIAmp DNA Blood Mini Kit (Qiagen, USA). The final volume of DNA solution after each extraction was 50 μL. A real time PCR assay was set up in a volume of 30 μL. The following components were added to a PCR tube: 15 μL of 2× SensiMix™ Probe Master mix (Bioline, UK), 1.25 μL sense primer (420 nM), 1.25 μL antisense primer (420 nM), 0.625 μL Universal ProbeLibrary Probe # 162 (210 nM) (Roche, USA) and water to make up a total volume of 25 μl. Finally, 5 μL of the extracted serum DNA was added. Primers (sense 5-tcaaacgatggacgtgaaac-3; antisense 5-ttcatgggtcgcttgacac-3; 71 bp) and a probe specific for a porcine male sex-determining region Y (SRY) were generated with Probe Finder software (Roche, USA). As an amplification control, we used a PCR specific for porcine zinc finger X-chromosomal gene (ZFX), which is present in both male and female samples. Primers (sense 5-tgagttggtttgtgaacatgaat-3; antisense 5-cccttacagtgtactggtatttcaga-3; 91 bp) and probe #114 specific to ZFX were generated with Probe Finder software (Roche, USA). The ZFX PCR protocol was identical to the SRY PCR protocol; only 2 μL (700 nM) of each primer was used. The real time PCR was performed using a Step-One™ Real-Time PCR system (AppliedBiosystems, USA). Identical thermal profiles were used for both the SRY and the ZFX reactions: denaturation for 15 min at 95°C, followed by 45 cycles of 95°C for 15 s (denaturation) and 60°C for 1 min (annealing and elongation).

For PCR validation, DNA from randomly selected male and female fetal sera was tested with SRY and ZFX PCRs. Afterwards, PCR products were subjected to gel electrophoresis to ensure the correct size of the amplicons (Additional file 1). The specificity of the SRY PCR was validated by testing genomic DNA extracted from male or female swine ear skin; only porcine male DNA was amplifiable (Additional file 1).

Following validation of PCR analyses, female fetal and dam samples were analyzed. Each sample was analyzed in five and two-three replicates for SRY and ZFX, respectively, in the same plate. Positive and negative controls (DNA extracted from male sera and multiple negative water blanks, respectively) were included in every PCR run. Fluorescence data calculated by the Step-One™ Real-Time PCR system were collected for each well and a sample was considered positive for SRY or ZFX when there was an exponential increase in fluorescence during the PCR amplification. Randomly selected samples were also subjected to gel electrophoresis during the experiment, to ensure the correct size of the amplicons.

Strict precautions were taken to prevent PCR contamination. Separate DNA isolation kits were used for female and male samples. DNA extractions and set up of PCR assays were performed under ultraviolet (UV) light-equipped safety hoods, with UV run for 1 h between experiments. Aerosol-resistant pipette tips and disposable gloves were always used. Reagent controls, with water instead of serum were included in every DNA isolation run. Controls were consistently negative in all experiments. Isolation of DNA, setup of PCR reactions, PCR assays and gel electrophoresis of PCR products were performed in separate locations.

A probe mapping to the porcine Y chromosome (BAC clone 428D8; SSCYp1.2 chromosome location) and a probe mapping to the porcine X chromosome (BAC clone 223 G10; SSCXq2. chromosome location) were used in sex-typing fluorescence in situ hybridization (FISH) [25-27]. Liver and lungs of five randomly selected male and five female fetuses from all dams were subjected to FISH analysis. From the I_70 _dam, liver and lungs of three and five randomly selected male and female fetuses, respectively, were subjected to FISH analysis. Three 5-7 μm-thick cryosections were made from each sample. These sections were cut 100 μm apart from each other in the tissue block. All sections were examined using a Zeiss Axioplan 2 fluorescence microscope (in 50 fields, 100× objective for a total magnification of × 1000).

All data for statistical analyses were obtained only from dams euthanized at 100 days of gestation (Table 1). PRRSV-inoculated dams, which were euthanized at 80 and 110 days of gestation, were excluded from statistical analyses because the gestation stage influences transplacental cell exchange [1]. All statistical tests were performed with the SigmaPlot11 software. A 95% confidence interval (*p *< 0.05) was applied for the statistical significance.

All non-inoculated dams and their fetuses remained PRRSV-negative throughout the entire experiment. All inoculated dams became viremic (10^2.3-4.8 ^TCID_50_/mL) and transplacental infection occurred in the five dams inoculated with PRRSV at 90 days of gestation (Table 2). Twelve to one hundred percent of the fetuses from these dams were viremic with titres ranging between 10^2.2 ^-10^7.3 ^TCID_50_/mL (Additional file 2 and 3). Virus-positive cells were observed in the internal organs of viremic fetuses by PRRSV-specific IF staining. In contrast, dam I_70_, inoculated at 70 days of gestation, was viremic (10^2.3 ^TCID_50_/mL), but no viremia or PRRSV-positive cells were detected in her fetuses.

**Table 2:** Results of PRRSV titration, SRY and ZFX PCR in dam sera.

Dam	**PRRSV titre log10 TCID_50_/ml of sera at the day of**...		**SRY PCR results at the day of**...		**ZFX PCR results at the day of**...	
	
	inoculation	euthanasia	inoculation	euthanasia	inoculation	euthanasia
I_70_	-	2.3	+	-	+	+
I_90_-1	-	4.8	-	+	+	+
I_90_-2	-	3.3	-	+	+	+
I_90_-3	-	3.3	-	-	+	+
I_90_-4	-	2.3	-	-	+	+
I_90_-5^†^	-	-	+	+	+	+
Con-1	-	-	-	-	+	+
Con-2	-	-	-	-	+	+
Con-3	-	-	-	-	+	+

PRRSV titres < 1.0 (detection limit) were considered to be negative. ^†^Serum from dam I_90_-5 was also tested at 100 days of gestation and was PRRSV-positive with a PRRSV titre of 2.6 log10 TCID_50_/mL.

A PCR assay to determine the gender of pigs has been previously described [28]. However, this test amplifies human DNA too, which increases the chance of false-positive results due to contamination during processing the samples. In the present study, probe-based PCR assays that specifically detect porcine SRY and ZFX were designed.

**Figure 1:**
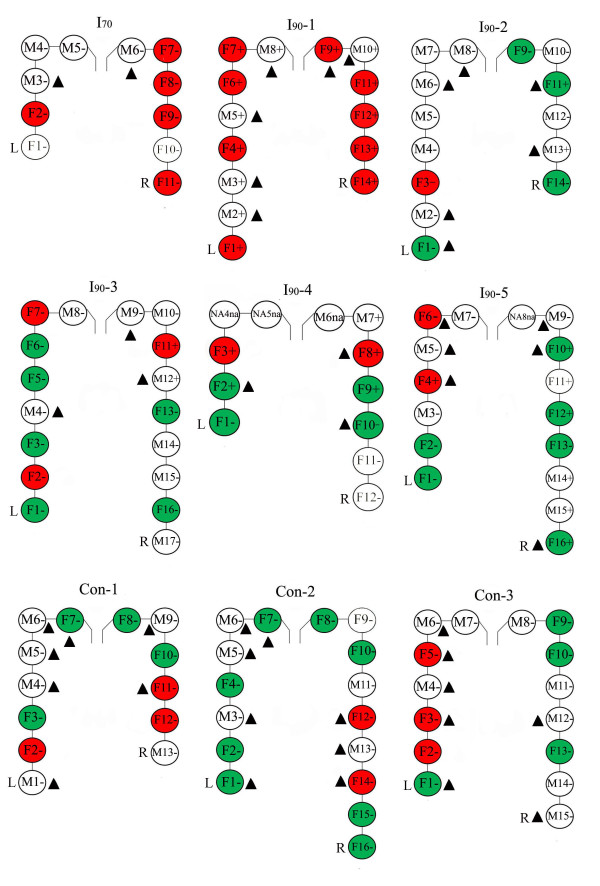
**Diagrams schematically represent the porcine uteri of the nine dams included in the study**. Fetuses were numbered starting with the fetus located next to the ovarian tip of the left uterine horn. L and R: left and right uterine horn, respectively. Each circle represents an individual fetus. F: female and M: male fetuses. "+" and "-" within the circles represent PRRSV-positive and -negative fetuses. Filled circles are female fetuses that were tested for the presence of male sex-determining region Y (SRY) in serum via PCR. Red and green circles are SRY-positive or -negative fetuses, respectively. Female fetuses with non-filled circles were not tested due to the lack of serum ▲: in these fetuses microchimeric cells were found. na: not available; due to mummification the determination of fetal gender and PRRSV infection status was not possible.

In total, 66 sera from female fetuses were tested in the SRY and ZFX PCRs. The results are summarized in Figure 1 and Additional file 2. SRY was detected in female fetal sera from both non-inoculated and PRRSV-inoculated dams. Non-inoculated and PRRSV-inoculated dams, had 20-43% and 20-100% of SRY-positive female fetuses, respectively. Male DNA was also detected in the maternal circulation of pregnant dams before and after infection (Table 2). All female fetal and maternal samples were amplifiable by the ZFX assay.

Microchimeric cells were detected via FISH in organs of fetuses from all nine dams (Figure 1, Additional file 2 and 3). Non-inoculated dams and dams with congenital PRRSV infection had 40-80% and 0-80% of female fetuses with male microchimeric cells and 80-100% and 0-100% of male fetuses with female microchimeric cells, respectively. In both cases, the number of fetuses which homed foreign cells did not differ significantly between the two dam groups (*p *> 0.05, tested with the Chi-square test and Fisher exact test, Additional file 4). Transplacental infection did not also influence the number of microchimeric cells within fetuses of both genders (*p *> 0.05, tested with the Mann-Whitney rank sum test).

**Figure 2:**
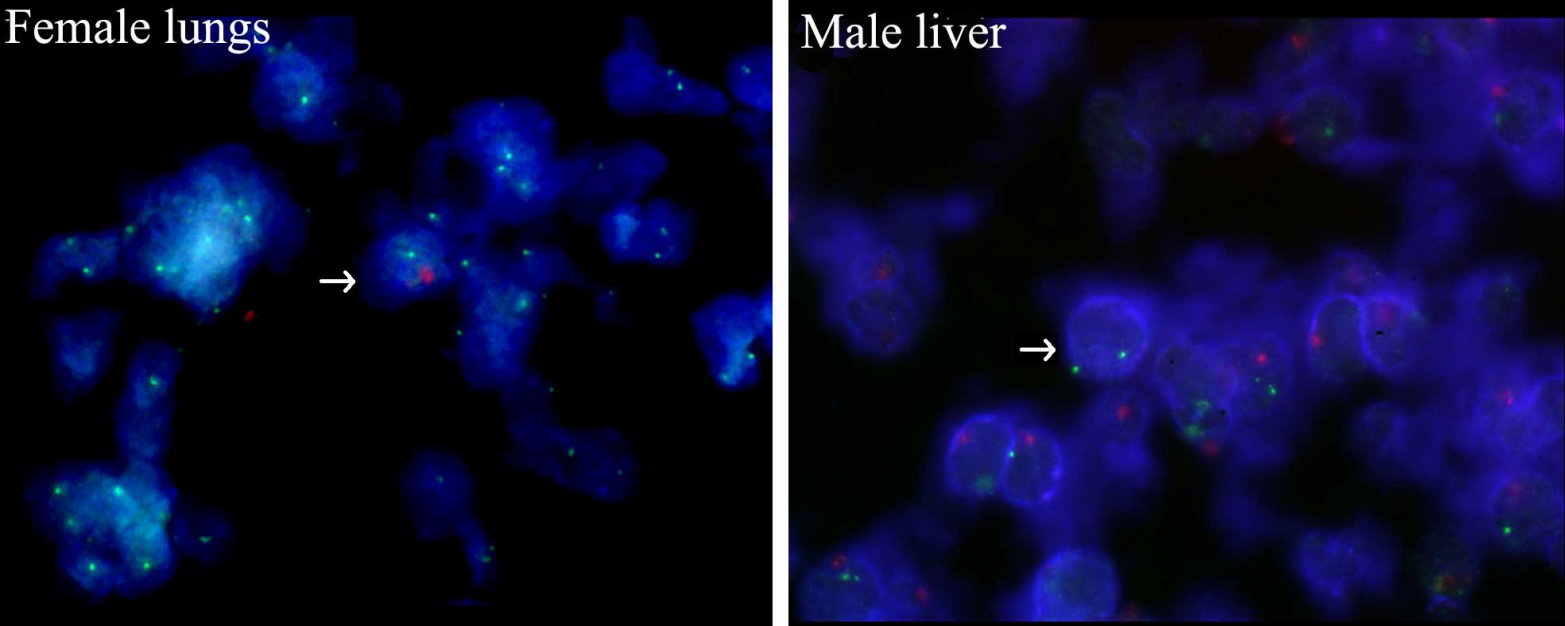
**Microchimeric cells found in female and male samples**. Red-dUTP and Green-dUTP (Abbott Molecular, USA) were used as fluorophores for the Y and X probes, respectively. FISH analysis on female lungs and male liver using probes for the X (green signal) and Y (red signal) chromosomes. Cell nuclei were counterstained with DAPI (blue). Arrows indicate microchimeric cells. FISH images of male and female cells were recorded using a Zeiss Axioplan 2 fluorescence microscope, a high-sensitivity integrated CCD camera and dedicated software (ISIS, MetaSystems, Germany).

Male (XY) cells were detected in 12 out of 30 tested female fetuses; 25 out of 30 male fetuses harbored female cells (*p *< 0.05, tested with the Chi-square test, Additional file 4). Female cells were found at higher numbers within organs of male fetuses versus the number of male cells within organs of female fetuses (*p *< 0.05, tested with the Mann-Whitney rank sum test). Representative images of microchimeric cells found in female and male samples are shown in Figure 2.

## Discussion

To the authors' knowledge, this is the first report of porcine transplacental DNA and/or nucleated cell trafficking in healthy as well as in PRRSV-infected dams and their fetuses. Only in one recent study, released during the preparation of this manuscript, human cells injected into porcine fetuses were detected in unmanipulated siblings [29].

In the present study, female cells were observed within tissues of male fetuses. These cells can be of female sibling and/or maternal origin. Concurrent invasion from female siblings and mother is also possible. The observations that the number of male microchimeric fetuses is significantly higher than the number of female microchimeric fetuses and that the number of female microchimeric cells within male tissues is significantly higher than the number of male microchimeric cells within female tissues is in agreement with the scenario of a double origin (sibling and maternal). Since PRRSV replication in the fetal implantation sites is restricted to Sn+/CD163+ macrophages [21,22] and as shown in the present study, porcine nucleated cells can migrate transplacentally, it is possible that PRRSV uses maternal susceptible macrophages as a vehicle to cross the uterine epithelium/trophoblast layers and reach fetal tissues. To further support this theory, the maternal/fetal origin of female macrophages within male fetuses should be demonstrated. The present experimental design did not allow to type microchimeric cells and to distinguish maternal from female fetal cells.

Male DNA and cells within sera and organs of female fetuses are most probably of male sibling origin. Porcine fetuses have individual fetal membranes and starting from 39-55 days of gestation, large central placental zones of the individual conceptuses are terminated by two extremities of the fetal sacs which include paraplacental and ischemic zones (necrotic tips) [30]. During pregnancy, adherence seems to occur between adjacent extremities of the fetal sacs [30]. Fetal cells may migrate between siblings through these extremities. After invading the neighboring sibling, fetal cells can degrade and release DNA and/or survive and engraft into tissues. From 60 days of gestation there are different degrees of adherences between fetal sac extremities within the uterus [30]. This may explain the difference in number of SRY-positive female fetuses between dams and in number of microchimeric cells within fetuses from the same dam. Some female fetuses that did not have in utero contacts with male fetuses also had SRY in sera and microchimeric cells within their tissues (Figure 1, Additional file 2). It looks like cells may pass from a male fetus through adjacent female siblings to more distant female fetuses.

In a study of Lager et al., selected porcine fetuses were inoculated in utero with PRRSV [31]. Afterwards, the virus could be isolated from inoculated as well as non-inoculated neighboring and more distant fetuses. It is very well possible that PRRSV is using sibling microchimeric cells to cross over the fetuses within the uterus. The proposed way of virus spread between mother and fetuses and in between siblings may not be only applied to PRRSV, but also to other porcine pathogens.

Interestingly, the percentage of PRRSV-positive animals was higher among female fetuses (50%) than among male fetuses (33%). This might suggest that the fetal gender is linked with the susceptibility of porcine fetuses to PRRSV infection. In line with this, previous observations in humans suggest that girls are at higher risk of in utero HIV infection than boys [32]. However, further research is necessary to determine if there is an actual direct connection between porcine fetal gender and susceptibility to PRRSV.

Microchimerism may benefit or compromise maternal and fetal health in humans [1,17-19]. Swine are recognized as a suitable animal model for human diseases based upon their comparative anatomy and physiology [33-35]. Further elaboration of microchimerism in swine will open new perspectives to design functional investigations to study the subject.

In the present study, it was demonstrated that PRRSV infection does not influence microchimerism. However, it is very well possible that PRRSV misuses maternal and sibling microchimeric cells during pregnancy to spread from mother to fetus and in between the fetuses. Further studies are needed to validate that maternal/fetal macrophages are spreading from dam to the fetuses and in between the fetuses, and that PRRSV misuses these cells to establish congenital infection.

## Competing interests

The authors declare that they have no competing interests.

## Authors' contributions

UUK conceived and designed the study, carried out PCR and participated in FISH, performed the statistical treatment of data and drafted the manuscript. WVB participated in FISH and helped in writing the manuscript. NVR developed and adapted FISH. CRG produced probes for FISH. HJN coordinated the work and helped in writing the manuscript. All authors read and approved the final manuscript.

## Supplementary Material

Additional file 1 **SRY and ZFX PCR assay validation**. During SRY and ZFX PCR assay validation, amplification products were subjected to gel electrophoresis to ensure the correct size of amplicons. SRY (amplicon size is 71 bp): (1) ladder; (2) fetal male serum DNA; (3 and 4) SRY-positive female fetal serum DNA; (5 and 6) SRY-positive dam serum DNA; (7) SRY-negative female fetal serum DNA; (8) SRY-negative dam serum DNA; (9) DNA from skin of male pig; (10) DNA from skin of female pig; (11) human serum DNA (a weak band of approximately 500 bp was observed in the human serum sample, but no positive signal was detected in the SRY real time PCR assay); (12) non template control. ZFX (amplicon size is 91 bp): (1) ladder; (2) male fetal serum DNA; (3) female fetal serum DNA; (4) dam serum DNA; (5) DNA form skin of male pig; (6) DNA from skin of female pig; (7) human serum DNA; (7) non template control. Click here for file

Additional file 2 **Virological, PCR and FISH findings in female fetuses**. Click here for file

Additional file 3 **Virological, PCR and FISH findings in male fetuses**. Click here for file

Additional file 4 **The statistical analyses of FISH data**. Click here for file
