# Supplementary figures and images for: Demonstration of microchimerism in pregnant sows and effects of congenital PRRSV infection
Source: Vet Res. 2012 Mar 16;43(1):19. doi: 10.1186/1297-9716-43-19 (PMC3368719; doi:10.1186/1297-9716-43-19)

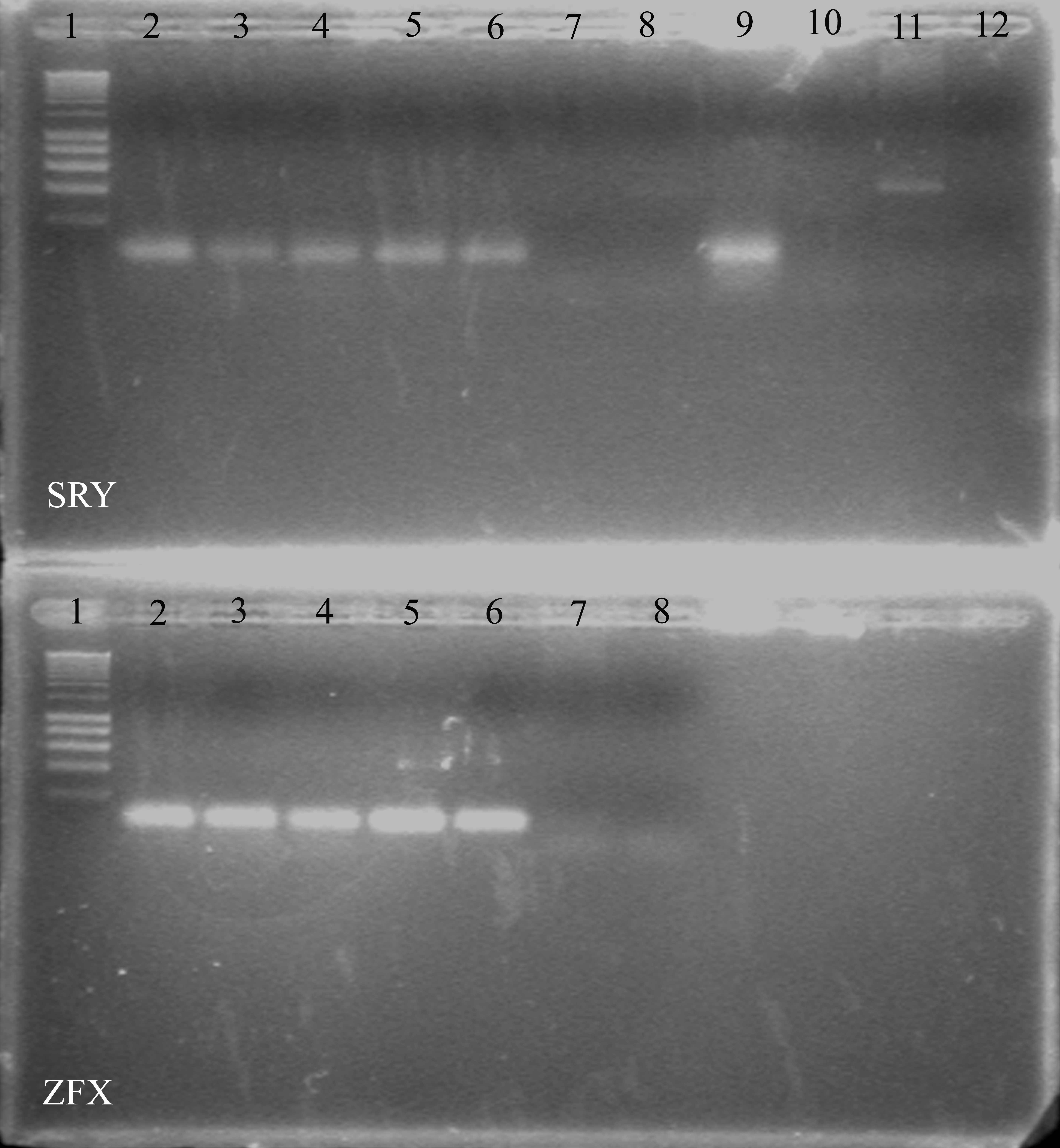

Supplement: Additional file 1 — SRY and ZFX PCR assay validation. During SRY and ZFX PCR assay validation, amplification products were subjected to gel electrophoresis to ensure the correct size of amplicons. SRY (amplicon size is 71 bp): (1) ladder; (2) fetal male serum DNA; (3 and 4) SRY-positive female fetal serum DNA; (5 and 6) SRY-positive dam serum DNA; (7) SRY-negative female fetal serum DNA; (8) SRY-negative dam serum DNA; (9) DNA from skin of male pig; (10) DNA from skin of female pig; (11) human serum DNA (a weak band of approximately 500 bp was observed in the human serum sample, but no positive signal was detected in the SRY real time PCR assay); (12) non template control. ZFX (amplicon size is 91 bp): (1) ladder; (2) male fetal serum DNA; (3) female fetal serum DNA; (4) dam serum DNA; (5) DNA form skin of male pig; (6) DNA from skin of female pig; (7) human serum DNA; (7) non template control. [file 1297-9716-43-19-S1.JPEG]
